# Meiotic nuclear movements in fission yeast are regulated by the transcription factor Mei4 downstream of a Cds1-dependent replication checkpoint pathway

**DOI:** 10.1111/gtc.12207

**Published:** 2014-12-10

**Authors:** Kun Ruan, Takaharu G Yamamoto, Haruhiko Asakawa, Yuji Chikashige, Hisao Masukata, Tokuko Haraguchi, Yasushi Hiraoka

**Affiliations:** 1 Graduate School of Frontier Biosciences, Osaka University1-3 Yamadaoka, Suita, 565-0871, Japan; 2 Advanced ICT Research Institute Kobe, National Institute of Information and Communications Technology588-2 Iwaoka, Iwaoka-cho, Nishi-ku, Kobe, 651-2492, Japan; 3 Department of Biological Sciences, Graduate School of Science, Osaka University1-1 Machikaneyama-cho, Toyonaka, 560-0043, Japan

## Abstract

In meiosis, the fission yeast nucleus displays an elongated morphology, moving back and forth within the cell; these nuclear movements continue for approximately 2 h before meiotic nuclear divisions. Meiotic DNA replication occurs in an early phase of the nuclear movements and is followed by meiotic prophase. Here we report that in mutants deficient in meiotic DNA replication, the duration of nuclear movements is strikingly prolonged to four to 5 h. We found that this prolongation was caused by the Cds1-dependent replication checkpoint, which represses expression of the *mei4*^*+*^ gene encoding a meiosis-specific transcription factor. In the absence of Mei4, nuclear movements persisted for more than 8 h. In contrast, overproduction of Mei4 accelerated termination of nuclear movements to approximately 30 min. These results show that Mei4 is involved in the termination of nuclear movements and that Mei4-mediated regulatory pathways link a DNA replication checkpoint to the termination of nuclear movements.

## Introduction

Meiosis is a particular type of nuclear division that is essential for sexual reproduction in eukaryotes. In meiosis, one round of DNA replication is followed by two consecutive rounds of chromosome segregation to generate four haploid gametes from a parental diploid cell. During this process, recombination between homologous chromosomes occurs to generate a recombined set of the haploid genome; this homologous recombination is crucial for the proper segregation of chromosomes.

Pairing and recombination of chromosomes in meiosis are promoted by a characteristic ‘bouquet’ arrangement of meiotic prophase chromosomes, in which telomeres form a cluster beneath the nuclear envelope to produce a bouquet-like arrangement of chromosomes (reviewed in Scherthan 2001; Harper *et al*. 2004; Hiraoka & Dernburg 2009). The fission yeast *Schizosaccharomyces pombe* provides the most striking example of this bouquet arrangement. In this organism, the nucleus elongates upon entering meiosis and moves back and forth between the cell ends, and telomeres remain clustered at the leading edge of the moving nucleus (Chikashige *et al*. 1994, 2007). This elongated nucleus is generally called the ‘horsetail nucleus’ (Robinow 1977), and its oscillation is termed ‘horsetail nuclear movement’. It has been shown that horsetail nuclear movements facilitate pairing and subsequent recombination by aligning homologous chromosomes along their entire length (Yamamoto & Hiraoka 2001; Ding *et al*. 2004, 2010). The horsetail nucleus also provides a unique opportunity to examine the structure of chromosomes in a defined orientation (Ding *et al*. 2006).

Faithful meiotic DNA replication is a prerequisite for transmitting genetic information from parents to offspring. In *S. pombe*, meiotic replication occurs approximately at the beginning of the horsetail stage (Bähler *et al*. 1993; Chikashige *et al*. 2004). For DNA replication, synthesis of deoxyribonucleotides (dNTPs) is necessary. dNTPs are converted from NTPs by ribonucleotide reductase (RNR). In *S. pombe*, RNR is a heterotetramer containing two large subunits of Cdc22 and two small subunits of Suc22 (reviewed in Nordlund & Reichard 2006). An RNR inhibitor Spd1 plays an important role in the regulation of RNR activity. Spd1 imports Suc22 to the nucleus and also binds to Cdc22 in the cytoplasm, compartmentalizing Suc22 and Cdc22, to inhibit RNR activity (Håkansson *et al*. 2006; Nestoras *et al*. 2010). Upon entry into S phase, or in the presence of DNA damage, Spd1 is degraded by a ubiquitin-dependent pathway involving the Pcu4-Ddb1^Cdt2^ E3 ubiquitin ligase and its associated COP9 signalosome complex, leading to the initiation of DNA replication or DNA repair (Liu *et al*. 2003, 2005; Nestoras *et al*. 2010). The COP9 signalosome complex consists of eight subunits (Csn1-8) and is responsible for deneddylation of cullin-based E3 ubiquitin ligases, which is required to protect cullins from degradation (Wei *et al*. 2008). Interestingly, null mutation in either *csn1*^*+*^ or *ddb1*^*+*^ is not lethal for mitotically growing cells, but results in meiotic S-phase arrest (Holmberg *et al*. 2005; Nestoras *et al*. 2010). This meiotic arrest is probably caused by an insufficiency of dNTPs, as the arrest can be suppressed by the deletion of the *spd1*^*+*^ gene (Holmberg *et al*. 2005). This fact may reflect a difference in the RNR activity requirements of mitotic and meiotic S phase (Grallert & Sipiczki 1991; Holmberg *et al*. 2005).

The checkpoint response to DNA damage differs in mitotic and meiotic cells. Whereas DNA damage activates the Chk1-dependent checkpoint in mitotic cells (Walworth *et al*. 1993), it is not activated in meiotic cells and DNA damage is repaired by recombination without activating checkpoint arrest (Pankratz & Forsburg 2005). Alternatively, in meiotic cells under conditions of dNTP insufficiency, S-phase arrest is mediated by the replication checkpoint kinase Cds1 (Murakami & Nurse 1999). When DNA replication is inhibited, activation of the Cds1-dependent replication checkpoint represses expression of the *mei4*^*+*^ gene (Ogino & Masai 2006). Mei4 is a meiosis-specific transcription factor in *S. pombe*; cells deleted for the *mei4*^+^ gene complete meiotic DNA replication but do not enter meiotic divisions (Horie *et al*. 1998). It is known that Mei4 regulates Mde2 to induce DNA double-strand break formation for homologous recombination, linking DNA replication to homologous recombination (Abe & Shimoda 2000; Miyoshi *et al*. 2012). Mei4 is also required for the expression of phosphatase Cdc25, which activates cyclin-dependent kinase (CDK), and is thereby essential for triggering meiotic nuclear divisions (Iino *et al*. 1995; Murakami-Tonami *et al*. 2007). Meanwhile, activation of Cds1 inhibits CDK activity by activating Mik1 and Wee1 tyrosine kinases (Murakami & Nurse 1999), thus preventing entry into meiotic nuclear divisions. In the absence of Cds1, lethal nuclear division occurs without completion of meiotic DNA replication (Murakami & Nurse 1999).

In this study, we report a new phenotype of *csn1* mutants and other mutants with stalled DNA replication. These mutants could complete mitotic S phase but arrested at meiotic S phase, exhibiting prolonged movement of the horsetail nucleus. We found that horsetail nuclear movements were regulated by Mei4 downstream of the Cds1-dependent DNA replication checkpoint. These results provide the first demonstration that Mei4-mediated regulatory pathways link a DNA replication checkpoint to the termination of nuclear movements.

## Results

### Aberrant nuclear morphology and movement in *csn1* mutants

**Figure 1:**
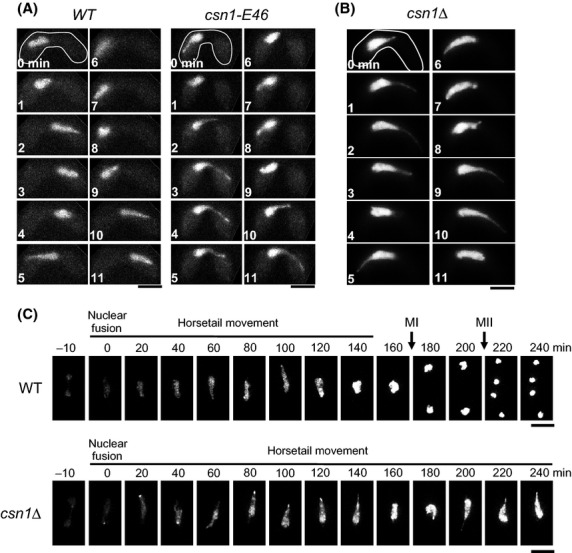
Phenotypes of *csn1* mutants. (A) Horsetail nuclear movement observed in living zygotes of wild-type and *csn1-E46* strains. Nuclei were stained with Hoechst33342. Numbers indicate the time in minutes. (B) Horsetail nuclear movement observed in the living zygote of the *csn1*Δ strain. The nucleus was labeled with Htb1 (histone H2B)-mCherry. Numbers indicate the time in minutes. (C) Progression of meiosis in wild-type and *csn1*Δ cells. The nucleus was labeled with Hht1 (histone H3)-mRFP. Numbers indicate the time elapsed since the beginning of horsetail movements. MI and MII indicate the timing of meiosis I and meiosis II, respectively. Scale bars indicate 5 μm.

We first isolated a mutant that completes conjugation and karyogamy but does not proceed to meiotic division. This mutant, named E46, proved to be allelic to *csn1*, which encodes a subunit of the COP9 signalosome (Zhou *et al*. 2001). The *csn1-E46* allele contained a base substitution (C to T) at position 25 that caused a non-sense mutation, suggesting that it may be a loss-of-function mutant. Both a *csn1* deletion strain (*csn1*Δ) and the *csn1-E46* mutant exhibited a characteristic nuclear morphology during horsetail nuclear movements: the tip of the horsetail nucleus, which protruded from the bulk of the nucleus, moved back and forth within the cell, but the bulk of the nucleus did not follow this movement (Fig.1A,B). In addition, we noticed that these mutants also exhibited prolonged horsetail nuclear movement. Figure1C shows an example of *csn1*Δ cells, in which the duration of horsetail nuclear movement was unusually prolonged. Because both mutants showed basically the same phenotype, we used the *csn1Δ* strain for further analyses.

### Meiotic DNA replication dynamics during nuclear movements of *S. pombe*

**Figure 2:**
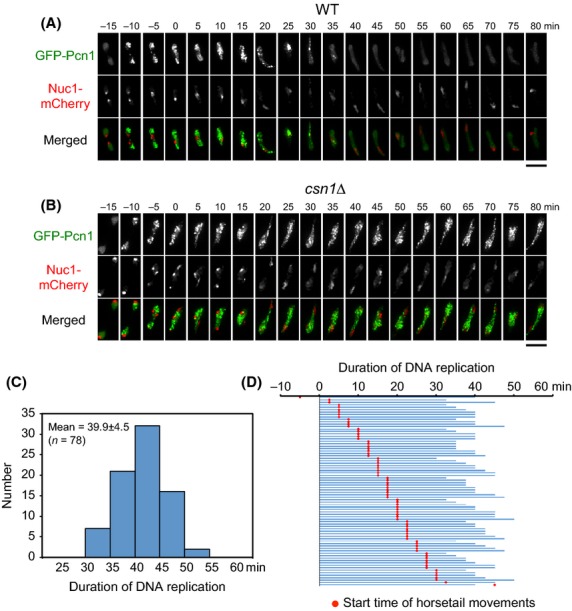
Time-lapse imaging of GFP-Pcn1 during meiosis. (A, B) Dynamics of GFP-Pcn1 (green) and Nuc1-mCherry (red) in wild-type cells (A) and *csn1*Δ (B) cells. Numbers indicate the time in minutes after the beginning of horsetail nuclear movements. Scale bars represent 5 μm. (C) Histogram of duration of DNA replication measured in 78 cells. (D) Start time of the horsetail movements (red spot) relative to the duration of DNA replication (blue line) for each of the 78 cells.

To confirm that the *csn1Δ* mutant has stalled DNA replication, we observed the meiotic DNA replication dynamics in living zygotes. To this end, we used PCNA tagged with GFP (GFP-Pcn1) as a marker for DNA replication, as it is known that the appearance of Pcn1 foci in the nucleus represents S phase in mitotic cells of *S. pombe* (Meister *et al*. 2007). As it has also been reported that DNA replication ends at the nucleolar rDNA region, in which ribosomal RNA is transcribed (Meister *et al*. 2007), we used the RNA polymerase I large subunit Nuc1 (Yamagishi & Nomura 1988) tagged with mCherry (Nuc1-mCherry) as a marker for the rDNA region to monitor the completion of S phase (Fig.2A).

A strikingly reproducible succession of GFP-Pcn1 patterns was observed in all examined cells. At the beginning of DNA replication, GFP-Pcn1 foci formed throughout the entire nucleus except for the rDNA region; the GFP-Pcn1 signals in the nucleus then became dim and accumulated only in the rDNA region at the end of DNA replication (Fig.2A). These results indicate that rDNA sequences are also replicated at the very end of S phase in meiotic cells. Based on the GFP-Pcn1 patterns, we determined when DNA replication occurs in zygotes, with the start and end of DNA replication being defined by the appearance and disappearance, respectively, of GFP-Pcn1 foci in the nucleus, whereas the beginning of horsetail nuclear movements was determined by the time of nuclear fusion (Fig.2A). The duration of wild-type meiotic DNA replication was 39.9 ±4.5 min as measured in 78 cells at 26 °C (Fig.2C), spanning the time when horsetail nuclear movements began (‘horsetail start’ in Fig.2D). In Fig.2D, the horsetail start time (marked by the red spot) is plotted relative to the DNA replication period (indicated by the blue line) for this sample. In virtually all of the cells examined (76 of 78), DNA replication began before the horsetail start and ended after it began; however, DNA replication ended before the horsetail start in one cell and began after it in another. The time points at which horsetail movement commenced in these cells were distributed throughout the DNA replication period, indicating that there is no direct link between DNA replication and nuclear fusion.

In *csn1*Δ cells, GFP-Pcn1 appeared at similar times to those recorded in wild-type cells. However, it remained in the nucleus throughout the prolonged nuclear movement period, and colocalization of GFP-Pcn1 and Nuc1-mCherry was not observed (Fig.2B), indicating that DNA replication was not completed during the observation period.

### Duration of nuclear movements is prolonged when cells are arrested at meiotic S phase

**Figure 3:**
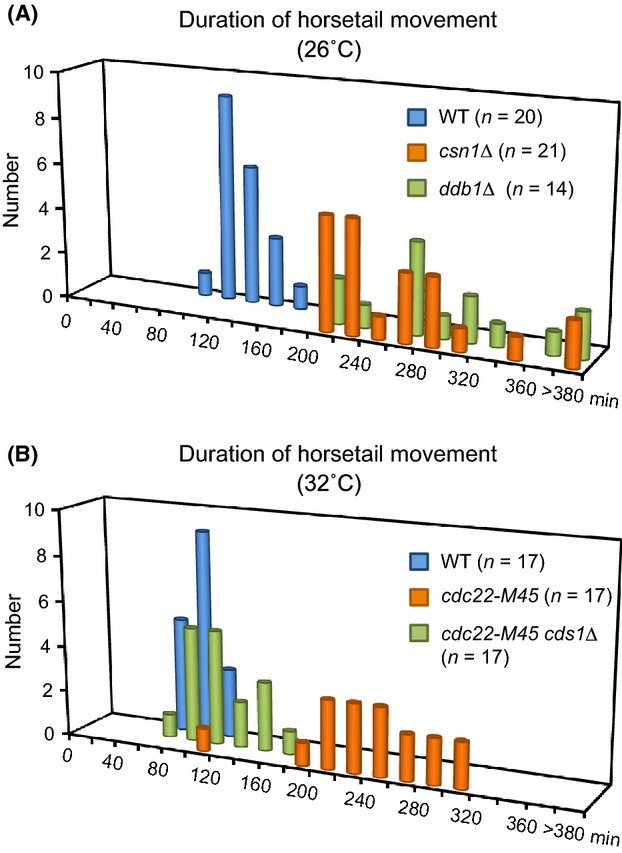
Prolongation of nuclear movements is dependent on Cds1. (A) Duration of horsetail nuclear movement graphed as a histogram for wild-type (blue), *csn1*Δ (orange), and *ddb1*Δ (green) cells at 26 °C. (B) Histogram showing the duration of horsetail nuclear movement in wild-type (blue), *cdc22-M45* (orange), and *cdc22-M45 cds1*Δ (green) cells at 32 °C.

To quantify the prolonged duration of horsetail nuclear movements associated with stalled DNA replication, we next measured the duration of nuclear movements in mutants. The average duration of nuclear movements was 121 ± 17 min in wild-type cells and was unchanged in most of the mutants that have been examined previously, including *taz1Δ, dhc1Δ,* and *rec12Δ* mutants (Ding *et al*. 2004). In contrast, the duration of the horsetail movements in *csn1*Δ and *ddb1*Δ cells was 262 ± 50 min and 289 ± 60 min, respectively, which is strikingly longer than that observed for wild-type cells (Fig.3A; Table1). We also examined a mutant for *cdc22-M45*, which is a temperature-sensitive allele of the *cdc22*^*+*^ gene encoding the RNR large subunit (Grallert & Sipiczki 1991). The average duration of the horsetail nuclear movements in *cdc22-M45* cells at a restrictive temperature of 32 °C was prolonged to 238 ± 50 min (Fig.3B), whereas the duration in wild-type cells was 85 ± 13 min at this temperature (Table2). Because *csn1*Δ, *ddb1*Δ and *cdc22-M45* mutants, which lead to insufficiency of dNTPs, all showed the same phenotype, we concluded that the prolongation of nuclear movements resulted from stalled DNA replication in meiosis.

**Table 1:** Duration of meiosis progression in various cell types at 26 °C

	Duration of horsetail movements (min)			End of horsetail movements to meiosis I (min)		
Strains and conditions	Mean	SD	*N*	Mean	SD	*N*
Wild-type	121	17	20	45	16	20
*csn1*Δ	262	50	21	No meiotic division		
*ddb1*Δ	289	60	14	No meiotic division		
*mei4*Δ	>480	ND	18	No meiotic division		
Wild-type with Mei4-GFP^OP^	32	27	26	71	24	23
*csn1*Δ with Mei4-GFP^OP^	32	35	24	No meiotic division		
Wild-type with Cdc25-GFP-NLS^OP^	97	26	28	47	14	21
*csn1*Δ with Cdc25-GFP-NLS^OP^	271	60	25	No meiotic division		
*mei4*Δ with Cdc25-GFP-NLS^OP^	96	46	35	42	12	22
*Prad21-cdc25*^***+***^	106	20	21	106	15	20
*Prad21-cdc25*^***+***^ with Mei4^OP^	21	22	21	84	18	20

*N*, number of cells examined; ND, not determined.

**Table 2:** Duration of meiosis progression in various cell types at 32 °C

	Duration of horsetail movements (min)			End of horsetail movements to meiosis I (min)		
Strains and conditions	Mean	SD	*N*	Mean	SD	*N*
Wild-type	85	13	17	48	17	21
Wild-type + HU	250	84	18	No meiotic division		
*cdc22-M45*	238	50	17	No meiotic division		
*cdc22-M45 cds1*Δ	109	25	17	54	30	15
Wild-type with Cdc25-GFP-NLS^OP^+HU	235	70	23	No meiotic division		
*mik1*Δ *wee1-50*	15	20	21	32	18	18
*mik1*Δ *wee1-50 *+* *HU	25	19	23	No meiotic division		
*mik1*Δ *wee1-50 mei4*Δ	22	22	23	69	28	18

*N*, number of cells examined.

We predicted that activation of the Cds1-dependent replication checkpoint extended the horsetail nuclear movements. To test this possibility, we used *cdc22-M45 cds1*Δ cells as the *csn1*Δ *cds1*Δ and *ddb1*Δ *cds1*Δ double mutants exhibited slow growth and low mating efficiency. Deletion of the *cds1*^*+*^ gene restored the duration of the horsetail nuclear movements to normal levels: the average duration for the *cdc22-M45 cds1*Δ cells was 109 ± 25 min at 32 °C (Fig.3B; Table2). These cells proceeded to meiotic nuclear divisions, being consistent with a previous report showing that *cds1*Δ cells treated with the RNR inhibitor hydroxyurea (HU) proceeded to meiotic nuclear divisions (Murakami & Nurse 1999). These results suggest that prolonged duration of horsetail nuclear movements is caused by activation of the Cds1-mediated replication checkpoint.

### Mei4 is required for termination of horsetail nuclear movement

**Figure 4:**
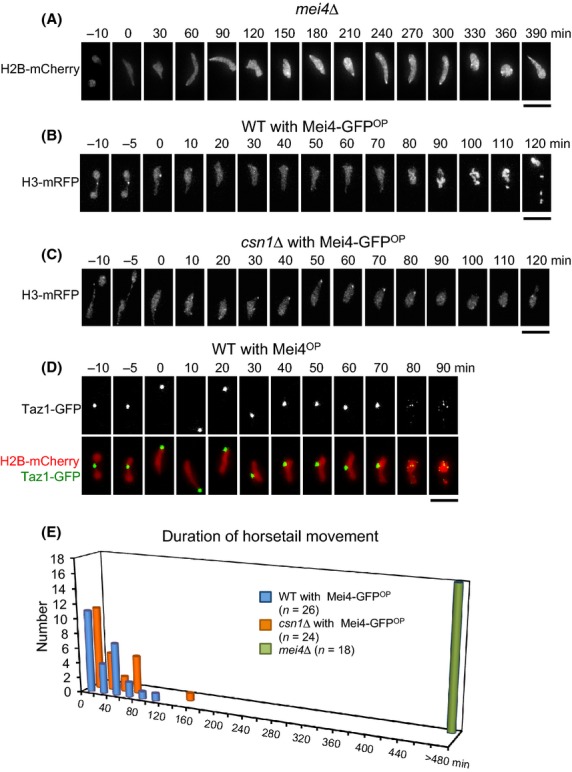
Nuclear movements are regulated by Mei4. (A) Horsetail nuclear movements in *mei4*Δ cells. Chromatin was labeled with Htb1 (histone H2B)-mCherry. Numbers indicate the time in minutes since the beginning of nuclear movements. The scale bar represents 5 μm. (B, C) Overproduction of Mei4-GFP in wild-type and *csn1*Δ cells. Chromatin was labeled with H3-mRFP. Numbers indicate the time in minutes since the beginning of nuclear movements. The scale bar represents 5 μm. (D) Dynamics of telomeres in wild-type cells with Mei4^OP^. Telomeres were labeled with Taz1-GFP (green), and chromatin was labeled with histone H2B-mCherry (red). The scale bar represents 5 μm. (E) Histogram of duration of horsetail nuclear movements in wild-type (blue) and *csn1*Δ (orange) cells overproducing Mei4-GFP.

As it is known that the Cds1-mediated DNA replication checkpoint suppresses Mei4 (Ogino & Masai 2006), we examined the effects of deletion and overproduction of Mei4 on horsetail nuclear movement. In cells lacking the *mei4*^*+*^ gene (*mei4Δ*), nuclear movements persisted for the whole observation period of over 8 h (Fig.4A,E). In stark contrast, overproduction of Mei4-GFP (Mei4-GFP^OP^) resulted in the rapid termination of horsetail nuclear movements in both wild-type and *csn1*Δ cells (Fig.4B, C and E). The average duration of nuclear movements was strikingly shortened to 32 ± 27 min and 32 ± 35 min in wild-type cells expressing Mei4-GFP^OP^ and in *csn1*Δ cells expressing Mei4-GFP^OP^, respectively (Table1). Importantly, the time from the end of horsetail nuclear movement to meiotic division in wild-type cells expressing Mei4-GFP^OP^ was extended to 71 ± 24 min from 45 ± 16 min in control wild-type cells (Table1). During this extended period, the nucleus remained in the elongated horsetail shape with clustered telomeres (Fig.4D). *csn1*Δ cells expressing Mei4-GFP^OP^ did not enter meiotic divisions. These results clearly indicate that horsetail nuclear movements are stopped downstream of Mei4.

### Mei4 terminates nuclear movements independently of Cdc25

**Figure 5:**
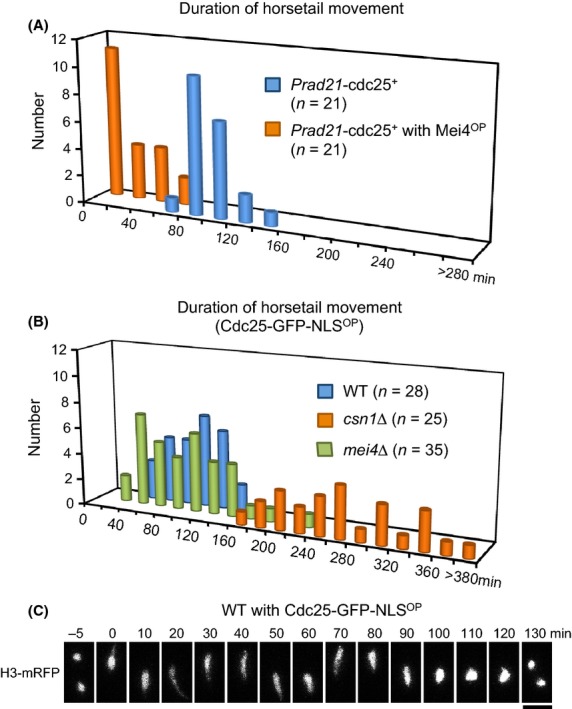
Cdc25 is dispensable for termination of nuclear movements. (A) Histogram of duration of horsetail nuclear movements in *Prad21-cdc25*^*+*^ cells with (orange) or without (blue) *mei4*^*+*^ overproduction. (B) Histogram of duration of horsetail nuclear movements in wild-type (blue), *csn1*Δ (orange), and *mei4*Δ (green) cells overproducing Cdc25-GFP-NLS. (C) Overproduction of Cdc25-GFP-NLS in wild-type cells. Chromatin was labeled with histone H3-mRFP. Scale bar represents 5 μm.

As Mei4 is an activator of *cdc25* transcription (Murakami-Tonami *et al*. 2007), we next examined the effect of depletion of Cdc25 on horsetail nuclear movement. Toward this end, we constructed a *cdc25*^*+*^ meiotic shutoff strain (referred to as *Prad21-cdc25*^*+*^), in which transcription of *cdc25*^*+*^ is under the control of the *rad21* promoter and is repressed during meiosis (Kitajima *et al*. 2004). The presence of reduced levels of Cdc25 in *Prad21-cdc25*^*+*^ cells was confirmed using GFP-tagged Cdc25; Cdc25-GFP signals were detected during meiotic nuclear divisions under the control of the native promoter, but no signals were detected under the control of the *rad21* promoter (data not shown). There was no significant difference in the duration of horsetail nuclear movements between wild-type and *Prad21-cdc25*^*+*^ cells, and overproduction of Mei4 in *Prad21-cdc25*^*+*^cells accelerated termination of horsetail nuclear movements (Fig.5A; Table1). These results indicate that Mei4 terminates nuclear movements independently of Cdc25.

### Cdc25 triggers entry to nuclear divisions

To further examine whether Cdc25 plays a role in the progression of meiosis, we examined the effect of overproduction of Cdc25. Cdc25-GFP-NLS was ectopically overproduced during meiosis under the control of the *nmt1* promoter and the 3′-UTR of the *rec8*^*+*^ gene. During vegetative growth, mRNA containing the 3′-UTR of the *rec8*^*+*^ gene is eliminated by the Mmi1-mediated RNA degradation system (Harigaya *et al*. 2006). Wild-type cells expressing Cdc25-GFP-NLS^OP^ exhibited nuclear movements for 97 ± 26 min and then entered meiotic division after 47 ± 14 min, a similar interval to wild-type cells (Fig.5B,C; Table1). Cdc25-GFP-NLS^OP^ rescued the prolongation of nuclear movements and meiotic arrest in *mei4Δ* cells (Fig.5B): *mei4Δ* cells expressing Cdc25-GFP-NLS^OP^ ceased nuclear movements after 96 ± 46 min, and the subsequent time to meiotic division was similar to wild type (42 ± 12 min, Table1). In contrast, Cdc25-GFP-NLS^OP^ did not rescue prolonged nuclear movements and meiotic arrest in *csn1*Δ cells: the duration of nuclear movements remained prolonged in *csn1*Δ cells expressing Cdc25-GFP-NLS^OP^ (271 ± 60 min), and these cells did not enter meiotic division (Fig.5B; Table1). These results indicate that Cdc25 overproduction bypasses the requirement for Mei4 in both termination of nuclear movements and entry to nuclear divisions, but does not overcome the DNA replication checkpoint. However, Mei4 overproduction overcomes the DNA replication checkpoint in termination of nuclear movements but not in entry to nuclear divisions (Fig.4E; Table1). Thus, the action of Cdc25 in response to the replication checkpoint is distinct from that of Mei4.

An important difference between cells over-expressing Cdc25 and Mei4 was the time interval from the end of nuclear movements to meiotic division: relative to control wild-type cells (45 ± 16 min), this interval was unchanged in wild-type cells expressing Cdc25-GFP-NLS^OP^ (47 ± 14 min) but extended to 71 ± 24 min in wild-type cells expressing Mei4-GFP^OP^ (Table1). In addition, unlike cells expressing Mei4-GFP^OP^, cells expressing Cdc25-GFP-NLS^OP^ did not exhibit the elongated nucleus after cessation of nuclear movements (compare Fig.5C with Fig.4B). These results suggest that Cdc25 triggers entry to nuclear divisions downstream of the replication checkpoint independently of Mei4.

**Figure 6:**
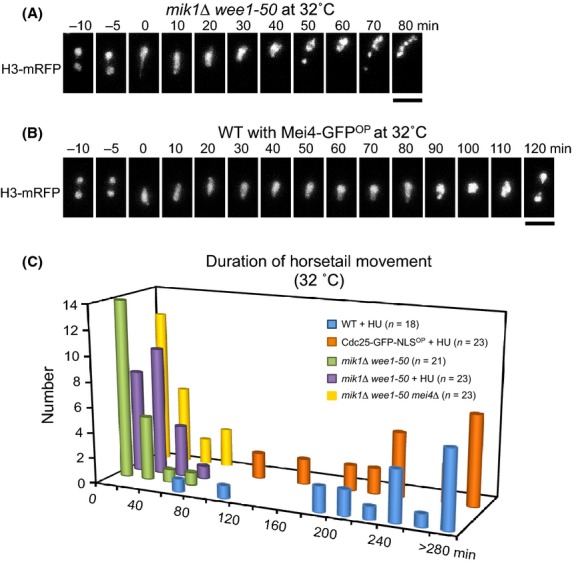
Nuclear movements in Mik1/Wee1 inactivation. (A) Horsetail nuclear movements in *mik1*Δ *wee1-50* at 32 °C. Chromatin was labeled with histone H3-mRFP. The scale bar represents 5 μm. (B) Horsetail nuclear movements in wild-type cells with Mei4-GFP^OP^ at 32 °C. Chromatin was labeled with histone H3-mRFP. Numbers indicate the time in minutes since the beginning of nuclear movements. The scale bar represents 5 μm. (C) Histogram of duration of horsetail nuclear movements at 32 °C for wild-type cells treated with HU (blue), Cdc25-GFP-NLS^OP^ cells treated with HU (orange), untreated *mik1*Δ *wee1-50* cells (green), *mik1*Δ *wee1-50* cells treated with HU (purple), and untreated *mik1*Δ *wee1-50 mei4*Δ cells (yellow).

To confirm the results obtained from the Cdc25 overproduction experiments, we investigated the effect of inactivation of Mik1/Wee1, which counteracts Cdc25 activation of CDK. Mik1/Wee1 phosphorylates Cdc2 on Tyr15 (Lundgren *et al*. 1991), therefore preventing Cdc25-mediated dephosphorylation at the same site. Thus, we measured the duration of horsetail nuclear movements in a *mik1Δ wee1-50* background at 32 °C (Lundgren *et al*. 1991). In *mik1*Δ *wee1-50* cells at 32 °C, the nucleus condensed to a spherical shape shortly after ceasing movement and entered nuclear division (Fig.6A), similar to the effect of Cdc25 over-expression (Fig.5C). In contrast, in cells over-expressing Mei4 at 32 °C, the nucleus remained elongated after cessation of nuclear movements until nuclear divisions commenced (Fig.6B), similar to the effect observed at 26 °C (Fig.4B). These results indicate that inactivation of Mik1/Wee1 triggers nuclear divisions, consistent with the results obtained from the Cdc25 overproduction experiments.

### Inactivation of Mik1/Wee1 overcomes DNA replication checkpoint

Cdc25 overproduction rescued the prolongation of nuclear movements in *mei4Δ* cells, but not in *csn1*Δ cells. This difference may result from activation of Mik1/Wee1 in response to the DNA replication checkpoint (Furnari *et al*. 1999). To test this possibility, we attempted to examine the effect of Mik1/Wee1 inactivation in a *csn1Δ* background. However, as the *csn1Δ mik1Δ wee1-50* mutation proved to be lethal, we used HU to inhibit dNTP synthesis instead deleting *csn1*^*+*^. As was the case for *csn1Δ* cells, the average duration of nuclear movements was prolonged to 250 ± 84 min in the presence of 15 mM HU and overproduction of Cdc25 did not rescue this effect (235 ± 70 min; Fig.6C and Table2). In contrast, in *mik1Δ wee1-50* cells at 32 °C, the duration of nuclear movements was shortened to 15 ± 20 min in the absence of HU or 25 ± 19 min in the presence of HU (Fig.6C and Table2). These results indicate that inactivation of Mik1/Wee1 overcomes the DNA replication checkpoint.

## Discussion

**Figure 7:**
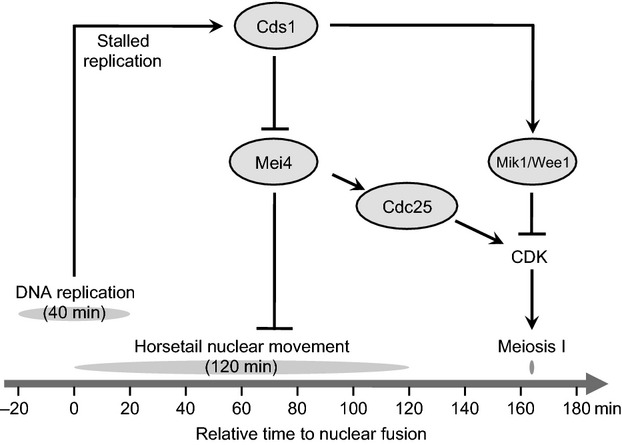
Regulatory networks linking DNA replication with nuclear movements. The transcription factor Mei4 is expressed specifically during meiosis and induces transcription of Cdc25. Cdc25 and Mik1/Wee1 counteract each other in the activation of CDK. Under DNA replication stress, the activation of Cds1 suppresses expression of Mei4, preventing the termination of horsetail nuclear movements. The activation of Cds1 also maintains the active state of Mik1/Wee1, preventing the entry to nuclear division. Overproduction of Mei4 or inactivation of Mik1/Wee1 bypasses the Cds1 checkpoint.

In this study, we have shown that abrogation of dNTP biosynthesis, which is caused by *csn1*Δ, *ddb1*Δ or *cdc22-M45* mutations or HU treatment, markedly prolongs the duration of horsetail nuclear movements and that this prolongation results from activation of the Cds1-dependent DNA replication checkpoint. We also determined the timing of DNA replication during meiosis in *S. pombe* for the first time and showed that DNA replication starts before nuclear fusion. Here we propose a model in which the Cds1-dependent DNA replication checkpoint coordinates the progression of meiosis through Mei4-mediated and CDK-mediated regulatory pathways (Fig.7). In the normal process of meiosis, the meiosis-specific transcription factor Mei4 terminates nuclear movements and induces Cdc25 expression. Expression of Cdc25 and inactivation of Mik1/Wee1 trigger nuclear divisions through CDK activation. Under DNA replication stress, the activated Cds1 checkpoint suppresses Mei4 to allow nuclear movements to continue, while at the same time maintaining the active state of Mik1/Wee1 to prevent entry to nuclear divisions. In the absence of Mei4, termination of nuclear movements or entry to nuclear divisions does not occur. Mei4 overproduction accelerates termination of nuclear movements while extending the time to nuclear divisions. Cdc25 overproduction or inactivation of Mik1/Wee1 triggers the entry to nuclear divisions without extension. Thus, Mei4 terminates nuclear movements, and CDK triggers nuclear divisions during the progression of meiosis.

### Mei4 is a major factor for the termination of horsetail nuclear movements

In wild-type cells, DNA replication is completed shortly after nuclear fusion and is followed by approximately 2 h of nuclear movement. The duration of nuclear movements has been shown to be constant in many previously examined mutants which were defective in telomere clustering (e.g., *taz1* or *rap1* mutants) (Ding *et al*. 2004). Likewise, the duration of nuclear movements is not affected by homologous recombination, as a *rec12* mutant also has a similar duration of nuclear movement to that of wild-type cells (Ding *et al*. 2004). Thus, it follows that the duration of nuclear movements is not determined by the completion of these events.

In this study, we report that the stalling of DNA replication in meiosis leads to prolongation of horsetail nuclear movements through activation of the Cds1-dependent replication checkpoint, which suppresses induction of Mei4. Our results show that depletion of Mei4 arrests cells at the horsetail stage, producing never-ending nuclear movements. Our results also show that overproduction of Mei4 accelerates termination of nuclear movements independently of Cdc25 in both wild-type and *csn1*Δ cells, suggesting that Mei4 is a major terminating factor.

Horsetail nuclear movements are driven by pulling forces generated by the dynein/dynactin complex along cytoplasmic microtubules (Ding *et al*. 1998; Yamamoto *et al*. 1999), and oscillatory movement is achieved by alternating activation and inactivation of the dynein/dynactin motor complex at each end of the cell (Yamamoto *et al*. 1999). A possible explanation for the observed Mei4-mediated termination of nuclear movements is that this alternating cycle is cancelled downstream of Mei4 expression, although the identity of direct effectors remains unknown.

### CDK activation triggers entry to meiotic nuclear divisions

In Mei4 overproduction, the nucleus remains in the horsetail stage after ceasing movements, as indicated by the elongated nucleus with clustered telomeres. In contrast, Cdc25 overproduction or Mik1/Wee1 inactivation leads to quick entry to nuclear divisions after the cessation of movements. This difference indicates that Mei4 terminates nuclear movements in the horsetail stage, whereas CDK aborts nuclear movements by driving nuclear divisions. A possible explanation for CDK-mediated abortion of nuclear movements is that cytoplasmic microtubules are reorganized to spindle microtubules upon entry to nuclear divisions (Kakui *et al*. 2013).

When the Cds1-dependent replication checkpoint is activated, Cdc25 overproduction is not sufficient for the abortion of nuclear movements, and Mik1/Wee1 inactivation is required, suggesting that Mik1/Wee1 dominates the counteractive Cdc25. Mik1/Wee1-mediated inhibition of CDK explains why overproduction of Cdc25 rescues defects in the termination of nuclear movements in *mei4*Δ cells*,* but fails to rescue these defects in *csn1*Δ cells. It should also be noted that overproduction of Cdc25 or inactivation of Mik1/Wee1 does not trigger entry to nuclear divisions when the Cds1 checkpoint is active, suggesting that entry to nuclear divisions is regulated by a separate pathway downstream of Cds1. This is consistent with a previous report that proposed the presence of a putative pathway, distinct from the Mik1/Wee1-mediated pathway, which is required for entry to meiotic divisions (Murakami & Nurse 1999).

Our results show, for the first time, a link between meiotic DNA replication and termination of horsetail nuclear movements. Under DNA replication stress, activation of the Cds1-dependent DNA replication checkpoint suppresses expression of the meiosis-specific transcription factor Mei4 and maintains the inactive state of CDK through Mik1/Wee1 to coordinate the progression of meiosis.

## Experimental procedures

### Strains and culture media

*Schizosaccharomyces pombe* strains used in this study are listed in Table S1 (Supporting Information). YES or YEA medium was used as complete medium, and EMM2 medium, containing nutritional supplements when necessary, was used as minimal medium. EMM2-N (EMM2 lacking a nitrogen source) medium and ME medium were used as sporulation media. All media were prepared as described in Moreno *et al*. (1991).

### Construction of plasmids and strains

Gene disruption for all described genes and gene tagging with GFP, mRFP, or mCherry for *nuc1*^+^ and *hht1*^+^ genes were carried out by the direct chromosomal integration method (Wach 1996; Bähler *et al*. 1998). Two-step PCR was used to amplify the integration cassettes. In the first round of PCR, the fragments were amplified from the 972 (*h*^−^, wild type) genome. For gene disruption, pFA6-kanMX6 (Bähler *et al*. 1998), pCR2.1-hph and pCR2.1-nat (Sato *et al*. 2005) were used to generate integration cassettes. For GFP, mRFP, or mCherry-tagging, pFA6a-GFP (S65T)-kanMX6 (Bähler *et al*. 1998), pFA6-mRFP-hphMX6 (Sato *et al*. 2005), or pFA6a-mCherry-hphMX6 (Funaya *et al*. 2012) was used to generate the GFP-, mRFP-, or mCherry-containing integration cassettes, respectively. Transformants were selected on YES plates containing 100 μg/mL G418 (Nacalai Tesque), 0.2 mg/mL hygromycin B (Wako), or 0.1 mg/mL nourseothricin (clonNAT, Werner BioAgents).

To observe chromatin in living cells, Htb1 (histone H2B) was tagged with mCherry. A 1.9 kb genomic fragment of the *hta1*^+^-*htb1*^+^ gene region was cloned by PCR, and the 3′-end of the *htb1*^+^ open reading frame (ORF) was fused to the mCherry gene in a plasmid harboring a selection marker gene. The 3′ UTR of the *htb1*^+^ gene was also cloned and inserted downstream of the mCherry genes for appropriate transcriptional termination. The resulting htb1-mCherry plasmid was integrated into the *aur1*^*+*^ gene locus. Transformants were selected on YES plates containing 0.5 μg/mL aureobasidin A (Takara).

To observe replication dynamics in living cells, GFP-Pcn1 under the control of the endogenous *pcn1*^*+*^ promoter was inserted into the *lys1* locus; the *pcn1*^+^ gene promoter, GFP gene, and *pcn1*^+^ gene were separately amplified by PCR and cloned into the *Pst*I-*Nde*I site of pCST3 using an In-Fusion HD Cloning kit (Takara), and the resulting plasmid was integrated into the *lys1* gene locus.

For overproduction of the Mei4-GFP gene product, the *mei4*^+^ ORF was integrated into the *Nde*I sites of pCST8, which harbors the *nmt1* promoter and GFP (Chikashige *et al*. 2006). The resulting plasmid was integrated into the *lys1* gene locus. Mei4-GFP is produced only in meiosis because the *mei4*^+^ transcript has the determinant of selective removal (DSR) sequence and is selectively eliminated under vegetative growth conditions (Harigaya *et al*. 2006). For overproduction of Cdc25-GFP-NLS, a DNA fragment encoding SV40 NLS was added downstream of GFP and the *cdc25*^+^ gene ORF was cloned. To repress mitotic expression of Cdc25-GFP-NLS, the DSR sequence for the *rec8*^+^ gene was also cloned downstream of the GFP-NLS, as described previously (Asakawa *et al*. 2010). Expression of Mei4-GFP and Cdc25-GFP-NLS under the control of the *nmt1* promoter was induced by removal of thiamine from the growth medium.

The *Prad21-cdc25*^*+*^ strain was generated as follows: a 1.5-kb DNA fragment carrying the *rad21* promoter (*Prad21*) was amplified by PCR and cloned into the *EcoR*I site of a pFA6a-natMX6 plasmid (Sato *et al*. 2005) using an In-Fusion HD Cloning kit (Takara) to produce a pFA6a-natMX6-*Prad21* plasmid. The resulting plasmid was used as a template to produce an integration cassette containing the *nat^r^* gene and *Prad21*. The resulting integration cassette was integrated into the promoter of *cdc25*^*+*^. Transformants were selected on YES plates containing 0.1 mg/mL nourseothricin.

### Isolation of the E46 mutant

Mutagenesis was carried out by ultraviolet (UV) irradiation to isolate mutants exhibiting abnormal nuclear morphology in meiosis. The CRL262 strain (*h*^*90*^ *pat1-114 leu1-32 lys1-131*), grown to log phase, was plated on YEA medium. The plated cells were irradiated with 254 nm UV (120 J/m^2^) and then incubated at 26 °C to form colonies. The survival rate was approximately 10%. Zygotic and azygotic ascus formation was examined for 12 000 viable clones, and 124 sporulation-deficient clones were isolated. Observation of meiotic nuclear morphology identified 33 clones exhibiting abnormal nuclear morphology, and backcross analysis identified four single mutants including E46.

### Fluorescence microscopy

A DeltaVision fluorescence microscopy system (Applied Precision), which is based on an Olympus wide-field IX71 fluorescence microscope equipped with an oil-immersion objective lens (Plan Apo 60×; NA = 1.4; Olympus) and CoolSNAP HQ2 CCD camera (Photometrics), was used to image the yeast cells. For time-lapse observation, living cells were mounted on 35-mm glass-bottomed culture dishes (MatTek) coated with 0.2 mg/mL soybean lectin (Sigma) and observed at 26 °C unless otherwise specified. A set of images of 15 focal planes at 0.5 μm intervals was taken at each time point. Images were deconvolved using the DeltaVision SoftWorx software (Applied Precision).

## Supporting Information

Table S1 Lists the strains used in this study and references their sources
