# Supplementary material for: Meiotic nuclear movements in fission yeast are regulated by the transcription factor Mei4 downstream of a Cds1-dependent replication checkpoint pathway
Source: Genes Cells. 2014 Dec 10;20(3):160–72. doi: 10.1111/gtc.12207 (PMC4359684; doi:10.1111/gtc.12207)
Supplement: Table S1 — Lists the strains used in this study and references their sources [file gtc0020-0160-sd1.docx]

Table S1. Strains used in this study.

|  | **Strain name** | **Genotype** | **Source** |
| --- | --- | --- | --- |
| **Figure 1** | 3E46-2b | *h^90^ leu1-32 ura4-D18 ade6-216 csn1-E46* | This work |
|  | 968 | *h^90^* | This work |
|  | RK478 | *h^90^* *ade6-210 leu1-32 lys1-131 ura4-D18 hht1^+^-mRFP-hph* | This work |
|  | RK1220 | *h^90^ ade6-210 leu1-32 lys1-131 ura4-D18 hht1^+^-mRFP-hph csn1∆::nat^r^* | This work |
|  | RK1280 | *h^90^ ura4-D18 leu1-32 ade6-210 lys1-131 csn1∆::hph aur1^r^::htb1^+^-mCherry* | This work |
| **Figure 2** | RK1216 | *h^90^ ade6-210 leu1-32 ura4-D18 lys1^+^::Ppcn1-GFP-pcn1^+^ nuc1^+^-mCherry-kan^r^* | This work |
|  | RK1217 | *h^90^* *ade6-210 leu1-32 ura4-D18 lys1^+^::Ppcn1-GFP-pcn1^+^ nuc1^+^-mCherry-kan^r^ csn1∆::hph* | This work |
| **Figure 3** | RK472 | *h^90^ ade6-216 ura4-D18 leu1-32 lys1-131 rec10^+^-GFP-HA-kan^r^ hht1^+^-mRFP-hph ddb1∆::nat^r^* | This work |
|  | RK478 | *h^90^* *ade6-210 leu1-32 lys1-131 ura4-D18 hht1^+^-mRFP-hph* | This work |
|  | RK768 | *h^90^ ade6-210 ura4-D18 leu1-32 lys1-131 cdc22-M45 cds1∆::ura4^+^ hht1^+^-mRFP-hph* | This work |
|  | RK770 | *h^90^ ade6-216 ura4-D18 lys1-131 leu1-32 cdc22-M45 hht1^+^-mRFP-hph* | This work |
|  | RK1220 | *h^90^ ade6-210 leu1-32 lys1-131 ura4-D18 hht1^+^-mRFP-hph csn1∆::nat^r^* | This work |
| **Figure 4** | RK988 | *h^90^ ade6-216 leu1-32 ura4-D18 mei4∆::ura4^+^ aur1^r^::htb1^+^-mCherry* | This work |
|  | RK1132 | *h^90^ ade6-210 leu1-32 lys1^+^::Pnmt1-mei4^+^-GFP ura4-D18 hht1^+^-mRFP-hph atb2^+^::kan^r^-Pnda3-CFP-atb2^+^* | This work |
|  | RK1180 | *h^90^ ade6-210 leu1-32 lys1^+^::Pnmt1-mei4^+^-GFP ura4-D18 hht1^+^-mRFP-hph atb2^+^::kan^r^-Pnda3-CFP-atb2^+^ csn1∆::nat^r^* | This work |
|  | RK1291 | *ade6-210 leu1-32 lys1+::Pnmt1-mei4^+^ ura4-D18 aur1^r^::htb1^+^-mCherry taz1^+^-GFP-kan^r^* | This work |
| **Figure 5** | RK1159 | *h^90^ ade6-210 leu1-32 lys1^+^::Pnmt1-cdc25^+^-GFP-NLS-Trec8 ura4-D18 hht1^+^-mRFP-hph atb2^+^::kan^r^-Pnda3-CFP-atb2^+^* | This work |
|  | RK1187 | *h^90^ ade6-210 leu1-32 lys1^+^::Pnmt1-cdc25^+^-GFP-NLS-Trec8 ura4-D18 hht1^+^-mRFP-hph mei4∆::nat^r^ atb2^+^::kan^r^-Pnda3-CFP-atb2^+^* | This work |
|  | RK1213 | *h^90^ ade6-210 leu1-32 lys1^+^::Pnmt1-cdc25^+^-GFP-NLS-Trec8 ura4-D18 hht1^+^-mRFP-hph atb2^+^::kan^r^-Pnda3-CFP-atb2^+^ csn1∆::hph* | This work |
|  | RK1426 | *h^90^ ade6-210 leu1-32 lys1-32 ura4-D18 hht1^+^-mRFP-hph cdc25^+^::nat^r^-Prad21-cdc25^+^* | This work |
|  | RK1449 | *h^90^ ade6-210 leu1-32 lys1^+^::Pnmt1-mei4^+^ ura4-D18 hht1^+^-mRFP-hph cdc25^+^::nat^r^-Prad21-cdc25^+^* | This work |
| **Figure 6** | RK478 | *h^90^* *ade6-210 leu1-32 lys1-131 ura4-D18 hht1^+^-mRFP-hph* | This work |
|  | RK1132 | *h^90^ ade6-210 leu1-32 lys1^+^::Pnmt1-mei4^+^-GFP ura4-D18 hht1^+^-mRFP-hph atb2^+^::kan^r^-Pnda3-CFP-atb2^+^* | This work |
|  | RK1187 | *h^90^ ade6-210 leu1-32 lys1^+^::Pnmt1-cdc25^+^-GFP-NLS-Trec8 ura4-D18 hht1^+^-mRFP-hph mei4∆::nat^r^ atb2^+^::kan^r^-Pnda3-CFP-atb2^+^* | This work |
|  | RK1459 | *h^90^ leu1-32 ura4-D18 hht1^+^-mRFP-hph mik1∆::ura4^+^ wee1-50 hht1^+^-mRFP-hph* | This work |
|  | RK1533 | *h^90^ leu1-32 ura4-D18 hht1^+^-mRFP-hph mik1∆::ura4^+^ wee1-50 hht1^+^-mRFP-hph mei4∆::kan^r^* | This work |
